# Recent Advances in Functionalized Carbon Quantum Dots Integrated with Metal–Organic Frameworks: Emerging Platforms for Sensing and Food Safety Applications

**DOI:** 10.3390/foods14122060

**Published:** 2025-06-11

**Authors:** Arul Murugesan, Huanhuan Li, Muhammad Shoaib

**Affiliations:** School of Food and Biological Engineering, Jiangsu University, Zhenjiang 212013, China; chemarul91@gmail.com (A.M.); shoaib_ju@hotmail.com (M.S.)

**Keywords:** CQDs@MOFs, heavy metals, pesticides, antibiotics, mycotoxins, pathogens, aromatic compounds

## Abstract

Carbon quantum dots (CQDs), with their excellent photoluminescence, tunable surface chemistry, and low toxicity, have emerged as versatile nanomaterials in sensing technologies. Meanwhile, metal–organic frameworks (MOFs) possess exceptionally porous architectures and extensive surface areas, and tunable functionalities ideal for molecular recognition and analyte enrichment. The synergistic integration of CQDs and MOFs has significantly expanded the potential of hybrid materials with enhanced selectivity, sensitivity, and multifunctionality. While several reviews have addressed QD/MOF systems broadly, this review offers a focused and updated perspective on CQDs@MOFs composites specifically tailored for food safety and environmental sensing applications. This review provides a comprehensive analysis of recent advances in the design, synthesis, and surface functionalization of these hybrids, emphasizing the mechanisms of interaction, photophysical behavior, and performance advantages over conventional sensors. Special attention is given to their use in detecting food contaminants such as heavy metals, pesticides, antibiotics, mycotoxins, pathogens, and aromatic compounds. Key strategies to enhance stability, selectivity, and detection limits are highlighted, and current challenges and future directions for practical deployment are critically discussed.

## 1. Introduction

Functionalized carbon quantum dots (CQDs) incorporated into metal–organic frameworks (MOFs) represent an innovative hybrid material with significant potential in sensing and food detection applications [1,2]. Carbon quantum dots, known for their remarkable photoluminescent properties, low toxicity, and biocompatibility, have been increasingly functionalized to enhance their interaction with target analytes [3]. When integrated with MOFs (crystalline materials characterized by their high porosity and made up of metal ions linked to organic ligands), functionalized CQDs enhance the system by providing features such as increased surface area, adjustable pore dimensions, and precise chemical reactivity [4]. The integration of CQDs with MOFs creates highly adaptable systems capable of detecting a wide range of food contaminants, such as heavy metals, pesticides, antibiotics, mycotoxins, microbial pathogens, and toxic aromatic compounds [5,6,7,8,9,10]. MOFs offer a stable support structure that helps preserve the integrity of CQDs while improving their sensitivity and selectivity by anchoring ligands that are specific to target molecules [11]. The resulting hybrid materials demonstrate enhanced luminescent properties, structural stability, and reusability, making them ideal candidates for sustainable, rapid, and non-destructive food analysis [12,13,14,15,16].

The synthesis methods for functionalized CQDs@MOFs composites can generally be categorized into in situ and post-synthetic approaches [17]. The in situ methods focus on simultaneously synthesizing MOFs and incorporating CQDs during the MOF crystallization process, ensuring strong integration [18]. In contrast, post-synthetic methods involve the modification of pre-synthesized MOFs by embedding functionalized CQDs, providing better control over the hybrid structure [19]. Recent advances in the field highlight innovative strategies such as solvothermal synthesis, microwave-assisted processes, and template-driven assembly, which optimize the integration of CQDs into MOF matrices [20,21,22,23]. These approaches improve the functional integration of CQDs with MOFs and allow for fine-tuning of the composites’ physical and chemical characteristics [24].

The structure and modification of these hybrid materials are crucial factors influencing their overall performance. Functionalization strategies can optimize the interaction between CQDs and MOF’s, improve charge transfer dynamics, and introduce specific binding sites for target molecules [25]. These hybrid systems have demonstrated potential in diverse sensing modalities, including fluorescence, electrochemical, and optical sensing, positioning them as strong contenders for swift, highly sensitive, and precise detection in intricate matrices [26]. These functionalized CQD-based MOFs exhibit enhanced optical and catalytic capabilities, along with strong adsorption capabilities, rendering them highly suitable for food safety-related applications [27]. Their function is to target a broad spectrum of contaminants including heavy metals, pesticides, antibiotics, toxins, microbial pathogens, and aromatic compounds using mechanisms such as fluorescence quenching, electrochemical detection, and adsorption-based removal [5,28,29,30,31,32]. Although several recent reviews have explored QD/MOF hybrids in diverse applications including the sensing of biomarkers, drugs, bioactive compounds, food additives, energy, optoelectronics, and biomedicine and have summarized their synthesis strategies, characterization methods, and progress across various fields [4,16,22,26], comprehensive analysis focusing specifically on functionalized carbon quantum dots (CQDs) being incorporated into MOFs for food safety and contaminant detection remains scarce. This review aims to fill that gap by providing a focused perspective on the design, synthesis strategies, functionalization approaches, and application-specific performance of CQDs@MOFs in food analysis. It highlights how the functionalization of CQDs enhances their synergistic interaction with MOFs, thereby improving selectivity, sensitivity, and stability in detecting a wide range of food-related contaminants.

**Figure 1:**
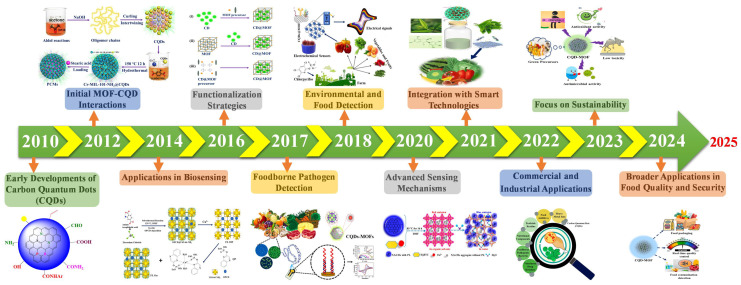
Timeline of CQDs@MOFs in sensing and food detection applications.

This review comprehensively examines the preparation, modification, and uses of CQDs@MOFs composites, highlighting the distinct characteristics that enable them to be effective in advanced sensing technologies. The discussion will highlight recent progress in developing these nanocomposites, including key advancements in fabrication techniques and their applications in the swift and precise identification of food contaminants, safeguarding food safety, and maintaining quality control. Timeline history of summary (2010–2024): The functionalization of CQDs with MOFs for sensing applications, particularly in food contaminant detection, has evolved significantly from early development to present-day innovations. With ongoing advancements in materials science and engineering, research continues to refine these hybrid materials for practical applications in food safety and quality control, as illustrated in Figure 1.

## 2. Synthesis Methods and Optimal Conditions for CQD–MOF for Food Safety Applications

The integration of functionalized CQDs with MOFs has become a key area of interest due to their potential in sensor development and food detection. The commonly employed methods for synthesizing CQDs@MOFs composites include encapsulation [33], post-synthetic modification [34], and in situ synthesis [24]. Recent advancements have also introduced additional synthesis approaches, such as top-down and bottom-up methods, to create these functional composites.

### 2.1. Top-Down Methods

Top-down approaches produce carbon quantum dots (CQDs) by breaking down bulk carbon materials, such as graphite or graphene, using techniques like chemical oxidation or laser ablation [23,35]. These techniques are cost-effective due to the use of abundant carbon materials and are scalable for industrial applications. They enable exact tuning of the surface characteristics of CQDs, which is crucial for integration with MOFs. Functionalization can occur during or after synthesis, improving CQDs@MOFs compatibility [4,36]. The resulting CQDs often carry diverse functional groups, enabling strong interactions with MOF structures. These interactions enhance the hybrid material’s optical, electronic, and catalytic behaviors [37,38,39]. Moreover, the top-down synthesis is a practical and effective strategy for CQDs@MOFs hybrid systems [40]. This approach supports the creation of advanced hybrid materials for sensing, catalysis, and energy storage. The versatility of CQDs combined with MOF modularity enables broad functional potential [41,42]. Top-down methods provide a flexible route to tailor CQDs for specific MOF-based applications.

#### Electrochemical Synthesis

Electrochemical synthesis is a highly efficient strategy for integrating functionalized CQDs with MOFs, effectively merging the photoluminescence, conductivity, and biocompatibility of CQDs with the modularity and large surface area of MOFs [43,44]. By applying a potential in a suitable electrolyte, this method facilitates the formation or functionalization of CQDs [45]. These CQDs can then be co-synthesized with or embedded into MOFs, forming composites with enhanced properties. MOFs act as scaffolds to anchor or encapsulate CQDs, while functional groups like -COOH and -NH_2_ promote stable binding. Moreover, electrochemical synthesis allows in situ growth of MOFs on CQDs and vice versa, ensuring strong adhesion [46]. Also, this method highlights their respective advantages and limitations in terms of particle size control, composite stability, and ease of functionalization for CQDs@MOFs, as illustrated in Table 1. Consequently, this approach ensures efficient integration and improved interaction, stability, and overall performance of the resulting materials.

**Table 1:** A concise comparison of synthesis methods in terms of particle size control, composite stability, advantages, and limitations, of CQDs@MOFs.

Method	Advantages	Limitations	Particle Size Control	Composite Stability	CQD@MOF Integration
Electrochemical	- Fast deposition - Good for thin films	- Needs conductive substrate - Limited MOF options	Moderate (~50–300 nm)	High (film-based)	CQDs co-deposited or anchored on MOF films
Hydrothermal/Solvothermal	- Strong interaction - Uniform embedding - One-pot synthesis	- Requires precise control of conditions - May damage CQDs at high temp	Moderate (100–500 nm)	Moderate to high	CQDs embedded during MOF crystal growth
Mechanochemical	- Solvent-free - Fast - Green and scalable	- Poor crystallinity - Irregular morphology - Limited size control	Nanocrystals to ~10 µm	Low (broad, irregular)	MOF crystals form around/with embedded CQDs
Microwave-Assisted Synthesis	- Rapid and uniform heating - Smaller MOF particles	- Risk of CQD damage if not optimized	Good (CQDs <10 nm; MOFs ~100 nm)	High	CQDs embedded or trapped in MOF matrix
Ultrasound-Assisted Synthesis	- Enhanced dispersion - Smaller particle size - Effective for hybrids	- CQD damage at high power - Scale-up is harder	Good (<100 nm CQDs; MOF ~100–300 nm)	High	CQDs mixed with MOF under cavitation-assisted nucleation
Layer-by-Layer (LbL) Assembly	- Precise placement - Thin film control - Multilayer structures	- Labor-intensive - Thin-film only	Excellent (nm-scale film thickness)	Very high	Alternating CQD/MOF layers or CQDs intercalated
Template-Assisted Synthesis	- Controlled morphology - Pore size tunable	- Template removal complexity - Limited CQD penetration	Variable (20 nm–µm range)	High	CQDs confined in templated cavities or structures

### 2.2. Bottom-Up Methods

Bottom-up methods involve assembling smaller building blocks into complex structures, and are widely applied in nanotechnology, materials science, and self-assembly [23,47,48,49]. In the context of functionalized MOFs incorporating CQDs, these approaches involve carefully assembling organic linkers with metal ions or clusters to create well-ordered crystalline structures [26,36]. Commonly used techniques include hydrothermal or solvothermal synthesis, solvent-free approaches, and mechanochemical methods, as well as microwave- and ultrasound-assisted synthesis [23,26,36,50,51]. These approaches allow precise control over CQD size, morphology, and surface properties, enabling effective integration into MOFs for specific functionalities [26,36]. They also ensure uniform CQD dispersion, essential for use in areas such as sensing, catalysis, and targeted drug delivery. A key advantage is the ability to introduce functional groups during synthesis, enhancing interactions between CQDs and MOFs [37]. This results in improved luminescence, conductivity, and catalytic performance. Additionally, the use of simple, scalable techniques supports large-scale production [38]. By adjusting precursors, conditions, and dopants, researchers can fine-tune optical and electronic properties [36,37,38,39]. Finally, the cost-effectiveness and compatibility of these methods make them ideal for developing robust CQD–MOF hybrids.

#### 2.2.1. Hydrothermal/Solvothermal Synthesis

Hydrothermal and solvothermal methods are common for synthesizing nanomaterials like CQD-integrated MOFs [23,36]. These techniques use water or organic solvents (e.g., ethanol or DMF) to dissolve precursors [33,52], enabling reactions in sealed reactors at 100–300 °C under pressure. This setup supports uniform crystal growth, high purity, and in situ functionalization [53,54]. It also underscores the respective advantages and limitations of the method in terms of particle size control, composite stability, and ease of functionalization for CQDs@MOFs, as summarized in Table 1. In CQDs@MOFs synthesis, CQDs or carbon sources (e.g., glucose and citric acid), metal salts (e.g., Zn^2+^ and Cu^2+^), and organic linkers (e.g., terephthalic acid and imidazole) are mixed in solution, heated to initiate growth, then filtered, washed, and dried [43,55]. The comprehensive summary of the reaction conditions, emission range, and size of quantum dots is presented in Table 2. These approaches ensure uniformity, high purity, and in situ functionalization by dissolving metal salts, organic linkers, and CQDs precursors in suitable solvents before heating in sealed reactors.

**Table 2:** Overview of synthesis methods and conditions for CQDs@MOFs composites.

S. No	CQDs@MOFs	Synthesis Methods	Emission Range (nm)	Size (nm)	Reference
1	BNCDs@Tb-MOF	Hydrothermal	450, 490 and 544	3	[38]
2	CQDs@ZIF-8	Hydrothermal	-	-	[56]
3	CDs@Eu-MOFs	Hydrothermal	365	3	[57]
4	CuO/Cu_2_O-CdS/HgS	Hydrothermal	-	-	[5]
5	MOF/CdTe QDs	Hydrothermal	425, 605	-	[58]
6	CDs@ZIF-90	Hydrothermal	453	-	[59]
7	E-CDs@ZIF-8	Hydrothermal	399 to 405	-	[60]
8	PEG-ZnSQD@ZIF-67	Green synthesis	420	-	[61]
9	CsPbBr_3_/HZIF-8	Room temperature	510	25	[62]
10	CDs@ZIF-8@SMIP	Hydrothermal	410–600	20	[6]
11	CDs@Cu-MOFs	Room temperature	430–600	-	[13]
12	CdTe QDs@ZIF-8	Room temperature	524–650	-	[63]
13	NH_2_-MIL-53 & N, P-CDs@MIP	Room temperature	360–438	-	[64]
14	MB@PApt-SP DNA@AZIS QDs@Ag-Pt NPs	Room temperature	-	-	[65]
15	M-TiO_2_-CdTe QDs/CdS QDs PEC	Room temperature	390	-	[66]
16	N-CDs@Eu-MOF@MIP	Room temperature	430–616	3	[67]
17	Fe-CDs/MOF-808 and Fe-CDs@MOF-808	Room temperature	~425	-	[11]
18	CD@UIO-66-NH_2_	Hydrothermal	425	5	[12]
19	N-CQDs@UiO-66-NH_2_	heated at 90 °C for 24 h	-	-	[27]
20	CdS-Sm-BDC-g-C_3_N_4_-5	Room temperature	-	-	[28]
21	CDs@Eu/UiO-67b	Hydrothermal	442–612	-	[20]
22	CdTe QDs@ZIF-8	Room temperature	521–672	-	[29]
23	CDs@UiO-66-NH_2_	Ultrasound	328	-	[68]
24	CD@MIP	Kettle Reflux	450	5	[69]
25	Ce, N-CDs@ZIF-67@MIP	Room temperature	445	-	[70]
26	His-GQDs-Ser@MOF	Room temperature	460–618	5	[71]
27	g-CDs@UiO-66	Stirred for 12 h at 60 °C	446–530	-	[72]
28	Co-CD/PMOF	Hydrothermal	350–450	-	[30]
29	MP QDs@ZIF-8	Room temperature	528	21	[8]
30	Antibody/MoS_2_/UiO-66-NH_2_	Microwave-assisted synthesis	-	-	[21]
31	NU66@QD-ICA	Room temperature	400–670	-	[73]
32	SQDs@MOF-5-NH_2_	Solvothermal method	645–755	-	[74]
33	N-GQDs/Au@Cu-MOF	Hydrothermal	-	-	[75]
34	GQDs/Cu-MOF	Ultrasonication	-	-	[76]
35	rGO-MWCNT/CS/CQD	Room temperature	-	-	[77]
36	DP-CDs/TiO_2_	Hydrothermal	520–420	-	[78]
37	[Zn(HCOO)_3_][C_2_H_8_N]/PEG and N-CQDs@[Zn(HCOO)_3_][C_2_H_8_N]/PEG	Hydrothermal and Room temperature	-	-	[31]
38	CD-Ab-COF	Room temperature	365	-	[9]
39	CDs@MIL-53(Fe)-NO_2_	Microwave-assisted synthesis	453	-	[79]
40	CDs-MFMIPs	Room temperature	400–600	-	[80]
41	CDs@ZIF-7	Room temperature	-	-	[81]
42	CDs@HKUST-1	Hydrothermal	-	-	[82]
43	CDs@MOF-5@Rh-6G	Hydrothermal	365, 435–560	-	[10]
44	BYCDs@ZIF-8	Room temperature	365, 440–565	-	[83]
45	CDs&ZIF-8@MIPs	Room temperature	-	-	[84]
46	N-GQDs@IRMOF-1@MIP	Room temperature	-	-	[85]
47	AgMOF@N-CD	Room temperature	-	-	[86]
48	B-CDs/P-CDs@ZIF-8	Room temperature	440–510	-	[87]

**Abbreviations: BNCDs**—boron and nitrogen carbon dots; **Tb**—terbium; **MOF**—metal–organic framework; **PEG**—polyethylene glycol; **QDs**—quantum dots; **ZIF**—zeolitic imidazolate framework; **CDs**—carbon dots; **SMIP**—surface molecularly imprinted polymer; **Ag-Pt NPs**—silver–platinum nanoparticles; **PEC**—photoelectrochemical; **M-TiO_2_**—metal-doped titanium dioxide; **BDC**—benzene-1,4-dicarboxylate; **Sm**—samarium; **CD**—carbon dot; **MIP**—molecularly imprinted polymer; **Ce, N-CDs**—cerium, nitrogen co-doped carbon quantum dots; **His-GQDs**—ser-histidine and serine-functionalized graphene quantum dots; **g-CDs**—green carbon dots; **Co-CD**—cobalt-doped carbon dots; **PMOF**—peroxidase metal–organic framework; **MP QDs**—methylamine perovskite quantum dots; **MoS_2_**—molybdenum disulfide; **ICA**—immunochromatographic assay; **SQDs**—sulfur quantum dots; **N-GQDs**—nitrogen doped graphene quantum dots; **GQDs**—graphene quantum dots; **MWCNTs**—multi-walled carbon nanotubes; **rGO**—reduced graphene oxide; **CS**—chitosan; **CQD**—carbon quantum dot; **DP-CDs**—*Diplocyclos palmatus* leaf extract-derived green-fluorescence carbon dots; **N-CQDs**—nitrogen-doped carbon quantum dots; **COFs**—covalent organic frameworks; **Ab**—antibody; **MFMIPs**—magnetic covalent organic frameworks molecularly imprinted polymers; **Rh-6G**—rhodamine 6G; **BYCDs**—blue and yellow emitting carbon dots; **IRMOF-1**—zinc metal–organic framework; **AgMOFs**—silver metal–organic frameworks; **N-CDs**—nitrogen-doped carbon quantum dots; **B-CDs**—boron-doped carbon dots; and **P-CDs**—phosphorous-doped green emitting carbon dots.

#### 2.2.2. Mechanochemical Synthesis

Mechanochemical synthesis is an environmentally friendly, solvent-free approach that relies on mechanical actions such as grinding or milling to initiate and promote chemical reactions. It is well-suited for producing carbon quantum dot (CQD)/metal–organic framework (MOF) composites due to its simplicity and ability to create unique materials [34,54,88]. Mechanical energy breaks and reforms chemical bonds without solvents [89], though small liquid additives (LAG) can enhance reactivity. Unlike traditional methods, it allows rapid, room-temperature synthesis with lower environmental impact [90,91,92,93]. Typically, CQD precursors like citric acid or glucose are combined with metal salts (e.g., ZnO or CuO) and organic linkers in a ball mill to form CQDs@MOFs, followed by washing and drying [94,95]. This technique is simple, energy-efficient, and capable of producing tailored functionalized materials, making it a promising strategy for advanced material synthesis. It also highlights the respective advantages and limitations in terms of particle size control, composite stability, and ease of functionalization for CQDs@MOFs, as summarized in Table 1.

#### 2.2.3. Microwave-Assisted Synthesis

Microwave-assisted synthesis involves the use of microwave radiation to quickly heat polar and ionic compounds by transforming electromagnetic energy into evenly distributed thermal energy [26,33,34,36,37]. This accelerates nucleation and growth, making it ideal for fabricating CQDs@MOFs with high energy efficiency, reduced synthesis time, and precise control over key parameters [94,95]. To prepare CQDs@MOFs, pre-synthesized CQDs and precursors like glucose or citric acid are dissolved in a solvent, followed by metal salts and organic linkers [33,36,43,96]. A comparative analysis of microwave- and ultrasound-assisted synthesis methods (Table 1 and Table 3) reveals their respective strengths and limitations in terms of particle size control, composite stability, and ease of functionalization. Microwave heating rapidly promotes crystallization and facilitates the uniform integration of CQDs, as demonstrated by Liu et al. in the formation of MIL-53(Fe)-NO_2_ using citric acid and ethylenediamine. Once the process is complete, the mixture is cooled, and the product is isolated via filtration and centrifugation, followed by washing and drying to yield a pure CQDs@MIL-53(Fe)-NO_2_ composite [79]. As summarized in Table 2, this microwave-assisted method offers a fast, energy-efficient route with fine control over material properties and crystallinity.

**Table 3:** Comparison of Microwave-Assisted vs. Ultrasound-Assisted Synthesis Methods.

Feature	Microwave-Assisted Synthesis	Ultrasound-Assisted Synthesis
Mechanism	Dielectric heating → rapid and uniform heating of reaction mixture	Acoustic cavitation → formation, growth, and implosion of bubbles that generate local hotspots
Reaction Time	Very short (minutes)	Short to moderate
Energy Input	Volumetric and uniform	Localized (at cavitation sites)
MOF Crystal Size Control	Good; can tune size by adjusting power/time	Moderate; harder to control due to stochastic cavitation
Quantum Dot (QD) Size Range	~2–10 nm (depending on precursor and time)	~3–15 nm, wider size distribution often observed
Product Homogeneity	Typically, high	Often lower (depends on sonication uniformity)
MOF Distribution on Substrate	More uniform coating possible	Can cause partial aggregation or uneven loading
Scalability	Moderate scalability (needs special equipment for large scale)	Easier to scale but uniformity issues persist
Advantages	- Rapid synthesis - High crystallinity - Narrow QD size distribution - Better control of morphology	- Simple setup - Can enhance porosity - Facilitates in situ functionalization - Green solvent-friendly
Limitations	- Expensive equipment - Risk of hot spots if not controlled - Limited to polar solvents	- Broader size distribution - Possible structural damage - Less efficient for crystalline MOFs

#### 2.2.4. Ultrasound Synthesis

Ultrasound-assisted synthesis uses high-frequency sound waves to trigger chemical reactions, offering rapid processing, low energy use, and the nanoscale precision ideal for integrating CQDs into MOFs [26,33]. Cavitation, caused by collapsing bubbles, generates extreme conditions (~5000 K, ~1000 atm) [34,97], enhancing molecular interactions and accelerating reaction rates, typically under ambient temperature and pressure conditions. Microwave- and ultrasound-assisted synthesis methods each offer distinct advantages and limitations [36]. A comparative analysis of these approaches, summarized in Table 3 and Table 1, highlights differences in particle size control, composite stability, and ease of functionalization for CQDs@MOFs. For instance, Lin et al. used ultrasonication to embed GQDs into Cu-MOFs by sonicating graphene oxide mixtures and combining the GQDs with Cu precursors [76]. Similarly, Liu et al. synthesized CDs@UiO-66-NH_2_ by ultrasonically mixing carbon dots with MOFs [68], as summarized in Table 2. These methods enable efficient CQDs@MOFs integration with precise control and reduced energy demand.

#### 2.2.5. Layer-by-Layer (LbL) Assembly

Layer-by-layer (LbL) assembly is a bottom-up technique for creating multifunctional composite materials by layering materials with opposite charges and complementary properties. This method allows precise control over thickness, composition, and functionality [98,99,100], particularly when integrating CQDs and MOFs [33]. CQDs are synthesized through processes like hydrothermal or electrochemical methods and functionalized to facilitate interaction. MOFs, selected based on application, serve as complementary building blocks [34,54,68,76,79]. The LbL process involves immersing a substrate in a CQD solution, followed by a MOF precursor solution to promote MOF growth. Crosslinking agents can enhance stability and interactions, resulting in robust hybrid materials for sensing, catalysis, and environmental remediation [100,101,102]. This technique paves the way for the development of advanced materials for a wide range of applications, including sensing, catalysis, and environmental remediation. As summarized in Table 1, this method also highlights the respective advantages and limitations of CQDs@MOFs, particularly regarding particle size control, composite stability, and ease of functionalization.

#### 2.2.6. Template-Assisted Synthesis

Template-assisted synthesis is a controlled fabrication method that uses a pre-defined template to direct material growth and morphology, making it ideal for synthesizing CQDs@MOFs. This approach allows precise control over the size, shape, and distribution of CQDs within the MOF matrix [103,104]. It also underscores the respective advantages and limitations of various synthesis strategies in terms of particle size regulation, composite stability, and ease of functionalization for CQDs@MOFs, as summarized in Table 1. Templates made from solid materials like silica and polymers are later removed, creating pores and specific morphologies, while surfactants, micelles, and polymers guide the assembly and decompose post-synthesis [19,105,106,107]. Typically, nanoparticles, nanofibers, or hollow spheres are used as templates, and CQDs are deposited onto or within them via physical adsorption, chemical bonding, or in situ growth [108,109,110,111]. Functionalized CQDs with MOF precursors, such as metal salts and organic linkers, are introduced to allow the MOF to crystallize around or within the CQD-template complex. Afterward, the template is removed, resulting in a well-defined CQDs@MOFs composite [112,113]. All the synthesis methods for various CQDs@MOFs under different techniques and conditions are summarized in Table 2. This approach enables accurate manipulation of both morphology and structure, facilitating the creation of high-performance materials with tailored properties and clearly defined architectures.

## 3. The Use and Properties of CQDs@MOFs

Carbon quantum dots (CQDs) are extensively studied in research owing to their remarkable photoluminescent characteristics, inherent biocompatibility, and adaptable surface functionalization potential. Their strong fluorescence, high quantum yields, and excellent photostability make them especially suitable for use in optical sensing applications [23,114,115,116,117]. Additionally, their non-toxic and environmentally friendly nature aligns with food safety requirements. By modifying functional groups, CQDs can achieve enhanced selectivity and stronger interactions with target molecules [35,118,119,120,121]. Similarly, MOFs offer high porosity, large surface areas, and tunable structures, enabling efficient and selective analyte capture [103,122]. Furthermore, the structures and properties of MOFs can be customized by modifying their metal nodes and organic linkers, allowing for precise tailoring to specific applications [104,112,123,124,125,126,127,128]. The integration of CQDs with MOFs has attracted growing interest for sensing and food detection, leveraging the complementary strengths of both materials [79]. This synergy results in composites with improved sensitivity and performance across diverse analytical applications [96]. The resulting CQDs@MOFs composites exhibit synergistic properties, further enhancing their effectiveness in sensing and food detection.

### 3.1. Enhanced Sensitivity and Selectivity

The integration results in enhanced sensing capabilities, attributed to the combined fluorescence of CQDs and the adsorption properties of MOFs. For instance, Jain et al. demonstrated the application of BNCDs@Tb-MOF as a fluorescent sensor for the highly sensitive and selective detection of Pd^2+^, utilizing a DNAzyme-based system [38]. Similarly, the use of an electrochemical sensor incorporating CQDs@ZIF-8 was reported for the detection of Cd^2+^, Cu^2+^, and Pb^2+^ ions, demonstrating excellent sensitivity and selectivity [56]. In addition, Guo et al. established a multifunctional fluorescent sensor using CDs@Eu-MOFs for the selective and sensitive detection of Hg^2+^ in water samples [57]. Furthermore, a MOF/CdTeQDs fluorescent sensor was designed for detecting Hg^2+^ and Cu^2+^, and showed excellent sensitivity and specificity toward different metal ions in actual sample analyses [58]. Moreover, Peng et al. reported a dual-functional fluorescent probe, CDs@ZIF-90, which exhibited highly sensitive and selective detection of Hg^2+^ and Al^3+^ [59].

As another example, Zhang et al. described that the composite carbon fiber membrane, NFE-CDs, exhibited strong blue fluorescence, contributing to its high sensitivity and selectivity. As a result, NFE-CDs were utilized as a fluorescent sensing platform for the detection of Cu^2+^ [60]. Similarly, Asadi et al. developed a PEG-ZnS QD@ZIF-67 composite that functions as a fluorescent sensor, allowing for the highly sensitive and selective detection of Cu^2+^ ions in aqueous samples [61]. Additionally, Ahmed et al. designed a CsPbBr_3_/HZIF-8 on–off–on fluorescence assay for the highly sensitive and selective detection of Cu^2+^ in melamine food samples [62]. Integrating CQDs with MOFs enhances metal ion sensing through improved fluorescence and detection, offering high selectivity and sensitivity for food safety applications.

### 3.2. Stability

MOFs provide a stable host for CQDs, enhancing composite robustness. For example, Liu et al. reported minimal signal fluctuation with a low RSD of 7.79%, confirming the CQDs@ZIF-8-modified electrode’s reliable performance in heavy metal detection [56]. Similarly, Zhang et al.’s investigation demonstrated high stability in zebrafish embryos over 0–5 h when detecting Cu^2+^ ions [60]. Furthermore, Asadi et al. developed a PEG-ZnS QD@ZIF-67 composite, which demonstrated long-term fluorescence stability for nearly 30 days while maintaining reliable detection of Cu^2+^ ions in water samples [61]. The fluorescent nanosensor showed good reproducibility, with intra- and inter-assay RSDs of 2.4% and 4.6% for detecting 420 nM Cu^2+^. In addition, Ahmed et al. developed a CsPbBr_3_/HZIF-8 composite stable under long-term open-air storage (~70% humidity). Ensuring long-term stability of CQDs and MOFs requires optimizing their composition, structure, and synthesis. Such optimization enhances reliable performance over time and under varying conditions [62]. These findings emphasize the role of composition, structure, and synthesis optimization in enhancing CQDs@MOFs stability, ensuring their long-term reliability for food safety monitoring.

### 3.3. Signal Amplification

The ability of MOFs to selectively accumulate target molecules significantly improves the sensitivity and specificity of nanoscale fluorescent sensors. For instance, Guo et al. developed a multifunctional fluorescent sensor, CDs@Eu-MOFs, which showed a fluorescence intensity shift from 430 nm to 614 nm when exposed to different concentrations of Hg^2+^ [57]. Similarly, the MOF/CdTeQDs sensor exhibited a color change at 605 nm under a 365 nm signal in response to varying concentrations of Hg^2+^ and Cu^2+^ [58]. Furthermore, Peng et al. reported a dual-mode signal using the CDs@ZIF-90 probe, with fluorescence intensity at 450 nm [59]. For example, Zhang et al. reported that the fluorescence intensity of NFE-CDs shifted from 390 nm to 405 nm in zebrafish embryos, and the fluorescence signal increased significantly upon the addition of Cu^2+^ ions [60]. Additionally, Asadi et al. demonstrated that the PEG-ZnS QD@ZIF-67 fluorescence probe had high adsorption capability, with its fluorescence at 420 nm being strongly quenched when Cu^2+^ ions were introduced, though the emission wavelength remained the same [61]. CQDs@MOFs composites offer a sensitive and selective platform for detecting food contaminants by combining CQD signal amplification with the MOFs tunable porosity. By integrating CQDs with MOFs, this platform leverages fluorescence, electrochemical, and colorimetric signals to enable rapid and sensitive detection of food hazards including heavy metals, pesticides, antibiotics, mycotoxins, pathogens, and aromatic organic compounds, thereby enhancing food safety monitoring.

## 4. Recent Progress in CQDs@MOFs-Based Sensing Applications

CQDs@MOFs composites have become innovative materials applied in sensor systems due to their synergistic properties, combining the optical advantages of CQDs and the structural versatility of MOFs. These composites enable highly sensitive, selective, and versatile sensing platforms suitable for food safety applications [6,13,129,130,131,132,133,134,135,136]. This review explores the development of CQDs@MOFs as advanced fluorescent probes for detecting food contaminants. By leveraging dual-mode sensing, these hybrid materials enhance detection sensitivity and selectivity through complementary fluorescence and colorimetric responses. Additionally, the enzyme-mimicking activity of CQDs@MOFs further improves detection capabilities by catalyzing reactions that amplify signal outputs. This multifunctional approach provides a rapid, reliable, and highly efficient method for identifying foodborne pollutants, ensuring improved food safety and quality control.

### 4.1. Enhanced Fluorescent Probes

Advances in CQDs@MOFs composites have significantly enhanced the performance of fluorescent probes in food detection applications. These composite materials integrate the strong photoluminescent properties and customizable nature of CQDs with the structural and functional advantages of MOFs, resulting in highly sensitive and selective sensing platforms [33,38,43,54]. For example, Pan and colleagues introduced a straightforward approach to fabricate a fluorescent probe, CDs@MOF@SMIP, for identifying chloramphenicol (CAP) in food samples. The probe demonstrated excellent sensitivity, achieving a low limit of detection (LOD) 0.0022 nM, and a linear fluorescence quenching response across CAP concentrations ranging from 0.323 μg L^−1^ to 8075.0 μg L^−1^. It showed excellent selectivity, sensitivity, and recovery rates (95.5–101.0%) in spiked food samples, with an RSD under 4.4%. This approach successfully detected trace amounts of CAP in food samples like milk, honey, and pork, demonstrating its strong potential for widespread use in food safety monitoring [6], as summarized in Table 4. In a similar vein, Yu et al. developed dual-emission fluorescent CDs@Cu-MOFs for detecting the pesticide thiophanate-methyl (TM) in food. The fluorescence intensity ratio (430 to 600 nm) showed a strong linear correlation with TM concentrations ranging from 0.0307 to 0.769 μmol L^−1^, with a low LOD of ~3.67 nM. The sensor effectively identified TM in fortified food samples recovery (93.1–113%) and real samples like apples, pears, and tomatoes, as summarized in Table 4 [13]. It allowed for visual detection by exhibiting a fluorescence color shift from blue to carmine, showcasing excellent sensitivity, selectivity, and suitability for practical food safety monitoring.

**Table 4:** Summary of CQDs@MOFs for detection of food contaminants including metal ions, pesticides, and antibiotics.

Contaminates	Food Samples	CQDs@MOFs	Sensors	Liner Range	LOD	Reference
**Heavy Metals/ions**						
Pb^2+^	Handpump water, Blue bird lake, Tap water, Chandigarh, NABI (Mohali), Manoli village water.	BNCDs@Tb-MOF	Fluorescent	0–1000 nM	5.97 nM	[38]
Pb^2+^, Cd^2+^ and Cu^2+^,	Tap water River water	CQDs@ZIF-8	Electrochemical	50 nM^−1^ μM	0.04 nM	[56]
Hg^2+^	Water	CDs@Eu-MOFs	Fluorescent	0–300 μM	0.12 nM	[57]
Hg^2+^	Rice, Peanuts and Water	CuO/Cu_2_O-CdS/HgS	Photoelectrochemical	0.5 pM to 2 μM	0.00011 nM	[5]
Hg^2+^ and cu^2+^	Lake water, Fruit juice and red wine	MOF/CdTe QDs	Fluorescence	-	0.6996 nM and 0.8268 nM	[58]
Al^3+^ and Hg^2+^	Yellow river water	CDs@ZIF-90	Fluorescent	1–200 μM for Al^3+^ and 0.05–240 μM for Hg^2+^	810 nM and 19.6 nM	[59]
Cu^2+^	School lake, Xuanwu lake, and Yangtze River waters	E-CDs@ZIF-8	Fluorescent		3.48 nM	[60]
Cu^2+^	Tap water	PEG-ZnSQDs@ZIF-67	Fluorescent	3 to 500 nM	0.96 nM	[61]
Cu^2+^	Tap water	CsPbBr_3_/HZIF-8	Fluorescent	3–500 nM for Cu^2+^ and 30–1500 nM for melamine	4.66 nM and 2.64 nM	[62]
**Pesticides**						
Chloramphenicol	Milk, Honey, and Pork	CDs@ZIF-8@SMIP	Fluorescent	0.323 μg L^−1^ (0.001 μM) to 8075.0 μg L^−1^ (25.0 μM),	0.0022 nM	[6]
Pesticide thiophanate-methyl	Apple, Pear, and Tomato	CDs@Cu-MOFs	Fluorescence	0.0307 to 0.769 μmol L^−1^	~ 3.67 nM	[13]
Chloramphenicol	Milk samples	M-TiO_2_-CdTe QDs/CdS QDs	Photoelectrochemical	1 to 140 nmol L^−1^	0.14 nM	[66]
Malathion	Tap water, and Soil samples	N-CDs@Eu-MOF@MIP	Fluorescent	1–10 μM	50 nM	[67]
Organophosphorus pesticides	Pakchoi and Water sample	Fe-CDs/MOF-808 and Fe-CDs@MOF-808	Fluorescent	0.001–360 μM and 0.01–100 μM	3.3 nM	[11]
Organophosphorus pesticide quinalphos	Tomato juice and Rice	OPCD@UiO-66-NH_2_	Fluorescent	0–16 μM	0.3 nM	[12]
						
carbendazim	Vegetables and Environmental samples	N-CQDs@UiO-66-NH_2_	Electrochemical	0.02–126 µM	20–126,000 nM and 5.8 nM	[27]
Malathion	Cabbage	CdS/g-C_3_N_4_/Sm-BDC MOF	Electrochemical (DPV)	3.0 × 10^−8^ to 15.0 × 10^−8^ M	7.4 nM	[28]
**Antibiotics**						
Tetracycline	Animal feeds	CdTe QDs@ZIF-8	Fluorescent/Colorimetry	0–70 μM and 0–1000 μM	15.5 nM 24.9 nM	[63]
Chlortetracycline	Milk	NH_2_-MIL-53 & N, P-CDs@MIP	Fluorescent and smartphone-integrated	0.06–30 μg·mL^−1^	28,787.88 nM 50,000.00 nM	[64]
Penicillin	Milk samples	MB@PApt-SP DNA@AZIS QDs@Ag-Pt NPs	Photoelectrochemistry, Electrochemiluminescence, and Fluorescence signals.	0.01 pg/mL^−1^ μg/mL (PEC), 1 pg/mL^−1^ μg/mL (ECL), and 1 pg/mL^−1^ μg/mL (FL),	0.0000034 nM, 0.00029 nM and 0.00047 nM	[65]
Ofloxacin and Tetracycline	Tap water and Chicken	Eu^3+^/CDs-modified UiO-67b	Fluorescent	0–60 µM and 0–10 µM	22/27 nM	[20]
Chlortetracycline	Basa fish and Pure milk	CdTe QDs@ZIF-8	Fluorescent	-	37 nM	[29]
Tetracycline and norfloxacin	Water, Milk and Soil samples	CDs@UiO-66-NH_2_	Fluorescent	-	150 nM and 870 nM,	[68]
Tetracycline	Milk samples	CD@MIP	Fluorescence	0–400 μmol L^−1^	590 nM	[69]
oxytetracycline	Milk	Ce, N-CDs@ZIF-67@MIP	Fluorescent	0.05–20 μg mL^−1^	15.13 nM	[70]
doxycycline	Milk	His-GQDs-Ser@MOF	Fluorescent	0.003–6.25 μM and 6.25–25 μM	1.8 nM	[71]
norfloxacin	Milk and Pork	g-CDs@UiO-66	fluorescent	1–8 μM	82 nM	[72]

**Abbreviations: BNCDs**—boron and nitrogen carbon dots; **Tb**—terbium; **MOF**—metal–organic framework; **PEG**—polyethylene glycol; **QDs**—quantum dots; **ZIF**—zeolitic imidazolate framework; **CDs**—carbon dots; **SMIP**-surface molecularly imprinted polymer; **Ag-Pt NPs**—silver–platinum nanoparticles; **PEC**—photoelectrochemical; **M-TiO_2_**—metal-doped titanium dioxide; **BDC**—benzene-1,4-dicarboxylate; **Sm**—samarium; **CD**—carbon dot; **MIP**-molecularly imprinted polymer; **Ce, N-CDs**—cerium, nitrogen co-doped carbon quantum dots; **His-GQDs**-ser-histidine and serine-functionalized graphene quantum dots; **g-CDs**—green carbon dots; nM—**nanomole**; ng/mL—**nanogram/milliliter**; LOD—limit of detection; and **μM**—micromolar.

### 4.2. Dual-Mode Sensing

CQDs@MOFs composites have emerged as promising materials for dual-mode sensing, combining fluorescence, electrochemical, and colorimetric techniques for more robust and reliable food safety monitoring [137,138,139]. These materials utilize the combined advantages of CQDs and MOFs, allowing for highly sensitive, selective, and versatile detection techniques capable of handling the intricate nature of food matrices [63,64,65]. For instance, Hui et.al. established a dual-mode sensing strategy using CdTe QDs@ZIF-8 for tetracycline detection, combining fluorescent and smartphone-based colorimetric sensors. The ZIF-8 framework inhibits the aggregation of quantum dots and produces a distinct green fluorescence at 524 nm; however, the presence of tetracycline suppresses the red fluorescence observed at 650 nm. This results in a distinctive butterfly-shaped spectrum, enabling ratiometric fluorescence detection with a linear range of 0–70 μM and an LOD of 15.5 nM. In addition, the smartphone-based colorimetric sensor leverages a red-to-green color change under UV light, achieving real-time detection over a broader range of 0–1000 μM with an LOD of 24.9 nM [63]. As a result, the constructed sensor demonstrated excellent practicality and significant potential for food safety applications, as summarized in Table 4.

Similarly, Zhang et al. established a dual-mode sensor, NH_2_-MIL-53 & N and P-CDs@MIP, for detecting chlortetracycline (CTC) in milk. The fluorescence-based sensor displayed a linear response to CTC ranging from 0.06 to 30 μg·mL^−1^, with an LOD of 28,787.88 nM. Applied to milk, it achieved a recovery rate of 88.73–96.28%. A smartphone-compatible device developed for sensing offered a cost-effective alternative to traditional spectrophotometers, with an LOD of 50,000.00 nM. The sensor demonstrated high selectivity, stability, and rapid detection, making it ideal for trace CTC detection in real samples, as summarized in Table 4 [64].

Furthermore, Li et al. induced a flexible multi-modal biosensor using a unique Ag-ZnIn_2_S_4_@Ag-Pt probe combined with a UiO-66 MOF for ultrasensitive penicillin detection. The probe, AZIS QDs@Ag-Pt NPs, exhibited excellent photoelectrochemical (PEC), electrochemiluminescence (ECL), and fluorescence (FL) properties. Upon binding to penicillin, the system generated strong multi-signal outputs. The detection platform showed wide linear ranges of 0.01 pg/mL^−1^ μg/mL (PEC), 1 pg/mL^−1^ μg/mL (ECL), and 1 pg/mL^−1^ μg/mL (FL), and LOD values of 0.0000034 nM, 0.00029 nM, and 0.00047 nM, respectively. It successfully detected penicillin in milk samples, highlighting its practical use, as summarized in Table 4. This innovative approach improves accuracy in food safety testing and health monitoring. Despite some fabrication challenges, such biosensors show great promise for real-time, sensitive contamination detection [65].

### 4.3. Enzyme Mimicry

The integration of CQDs with MOFs has paved the way for developing robust enzyme-mimicking systems (nanozymes) [140]. These composites replicate the catalytic activity of natural enzymes while offering superior stability, cost-effectiveness, and adaptability. CQDs@MOFs-based enzyme mimics are revolutionizing food detection applications by enabling precise, efficient, and sensitive analyses of contaminants and other food-related targets [30,79]. For instance, Yi et al. developed a dual-mode sensing strategy combining chemiluminescence and fluorescence using a Co-CD/PMOF nanozyme with strong peroxidase-like activity for detecting AFB1. This system demonstrated high sensitivity and was effective in real samples like canal water and milk. In the chemiluminescence mode, it achieved a detection range of 0.63–69.36 ng/mL and an LOD value of 0.217 ng/mL. In the fluorescence mode, with antibody-functionalized Co-CD/PMOF, it achieved a range of 0.54–51.91 ng/mL and an LOD value of 0.027 ng/mL. The study presents a rapid, sensitive, and reliable approach for environmental and food safety monitoring, as summarized in Table 5 [30]. Similarly, Liu et al. designed a dual-mode biosensor using CDs@MIL-53(Fe)-NO_2_, synthesized via a rapid microwave method. The carbon dots enhanced fluorescence and provided oxidase-like activity, enabling TMB oxidation without H_2_O_2_ and subsequent fluorescence quenching. This bifunctional nanozyme enabled sensitive detection of gallic acid, l-cysteine, and homocysteine, with LOD values of 17, 16, and 27 nM (fluorescence) and 62, 65, and 124 nM (colorimetric). The system was validated with green tea samples using a smartphone-based platform. Unlike typical CQDs@MOFs, it works without external stimuli [79]. This study highlights a new direction for efficient, dual-mode food safety biosensors, as summarized in Table 5.

**Table 5:** Summary of CQDs@MOFs for the detection of food contaminants, including mycotoxins, bacteria, and aromatic compounds.

Contaminates	Food Samples	CQDs@MOFs	Sensors	Liner Range	LOD	Reference
**Mycotoxins**						
Aflatoxin B1 (AFB1)	Canal water and liquid milk samples	Co-CD/PMOF	Chemiluminescence/ Fluorescence	0.63–69.36 ng/mL	0.217 ng/mL and 0.027 ng/mL	[30]
Aflatoxin B1	Corn	MP QDs@ZIF-8	Electrochemiluminescence	11.55 fg/mL to 20 ng/mL	0.0000035 nM	[8]
Aflatoxin M1	Milk samples	Antibody/MoS_2_/UiO-66-NH_2_	Electrochemical	0.2–10 ng/mL	0.06 ng/mL	[21]
Aflatoxin B_1_, Fumonisin B_1_, Deoxynivalenol, T-2 toxins, and Zearalenone	Cereals and Feed	NU66@QD-ICA	Fluorescent	-	0.04, 0.28, 0.25, 0.09, and 0.08 μg/kg.	[73]
Patulin (PAT)	Apple juice samples	SQDs@MOF-5-NH_2_	Fluorescent	-	0.000753 ng/mL	[74]
Patulin (PAT)	Apple juices	N-GQDs/Au@Cu-MOF	Electrochemical	0.001 to 70.0 ng/mL	0.0007 ng/mL	[75]
**Bacteria**						
*Staphylococcus aureus*	Tap water, Milk, *Lonicera japonica*, Urine, and Zhangjiang River.	GQDs/Cu-MOF	Electrochemical aptasensor	5.0 × 10^0^ to 5.0 × 10^8^ CFU·mL^−1^	0.97 CFU/mL	[76]
*Acinetobacter baumannii*	Skim milk powder	rGO-MWCNT/CS/CQD	Electrochemical aptasensor	10 to 1 × 10^7^ CFU/mL	1 CFU/mL	[77]
*Vibrio harveyi*	Shrimps	DP-CDs/TiO_2_	Fluorescent	-	-	[78]
*Escherichia coli*	-	[Zn(HCOO)_3_][C_2_H_8_N]/PEG and N-CQDs@[Zn(HCOO)_3_][C_2_H_8_N]/PEG	Fluorescent	-	-	[31]
*E. coli O157:H7*	Milk	CD-Ab-COF	Fluorescent	0 to 10^6^ CFU/mL	7 CFU/mL	[9]
**Aromatic compounds**						
Gallic acid (GA)	Green tea drink samples	CDs@MIL-53(Fe)-NO_2_	Colorimetric/Fluorescent		17, 16 and 27 nM	[79]
4-nitrophenol	Tap water, Fish and Shrimp meat	CDs-MFMIPs	Fluorescent	0.05–50 μM	17.44 nM.	[80]
Allura Red AC (AR)	Candy, Jelly, Strawberry flavored syrup, Pomegranate flavored drink, Energy drinks, Drink water, Commercial food colorant solution, and Carbonated beverages were determined.	CDs@ZIF-7	Fluorescent	0.30–7.00 nM	0.60 nM	[81]
Catechol	Tea samples	CDs@HKUST-1	Electrochemiluminescence	5.0 × 10^−9^ to 2.5 × 10^−5^ mol/L	3.8 nM	[82]
Curcumin	Cur in mustard, Curry, and red pepper powders.	CDs@MOF-5@Rh-6G	Fluorescent	0.1–5 μmol/L	15 nM	[10]
Glutathione	Grape and Cucumber	BYCDs@ZIF-8	Fluorescent	3–25 nM	0.90 nM	[83]
Malachite green (MG)	River water, Tap water, Deionized water and Aquaculture water	CDs&ZIF-8@MIPs	Fluorescent	20–180 nM	2.93 nM	[84]
Phenylureas	Tomato, Cucumber, Radish and Soybean milk	N-GQDs@IRMOF-1@MIP	Adsorbent	1.0–150 µg L^−1^	1.0 µg L^−1^	[85]
Trilobatin	Lithocarpus polystachyus Rehd	AgMOF@N-CD	Electrochemiluminescence	1.0 × 10^−7^ M to 1.0 × 10^−3^ M	5.99 nM	[86]
Triticonazole	Water and fruit juice samples	B-CDs/P-CDs@ZIF-8	Fluorescence	10–400 nM	4.0 nM	[87]

**Abbreviations: Co-CD**—cobalt-doped carbon dots; **PMOF**—peroxidase metal–organic framework; **MP QDs**—methylamine perovskite quantum dots; **MoS_2_**—molybdenum disulfide; **ICA**—immunochromatographic assay; **SQDs**—sulfur quantum dots; **N-GQDs**—nitrogen doped graphene quantum dots; **GQDs**—graphene quantum dots; **MWCNTs**—multi-walled carbon nanotubes; **rGO**—reduced graphene oxide; **CS**—chitosan; **CQD**—carbon quantum dot; **DP-CDs**—*Diplocyclos palmatus* leaf extract-derived green-fluorescence carbon dots; **N-CQDs**—nitrogen-doped carbon quantum dots; **COFs**—covalent organic frameworks; **Ab**—antibody; **MFMIPs**—magnetic covalent organic frameworks molecularly imprinted polymers; **Rh-6G**—rhodamine 6G; **BYCDs**—blue and yellow emitting carbon dots; **IRMOF-1**—zinc metal–organic framework; **AgMOFs**—silver metal–organic frameworks; **N-CDs**—nitrogen-doped carbon quantum dots; **B-CDs**—boron-doped carbon dots; **P-CDs**—phosphorous-doped green emitting carbon dots; **nM**—nanomole; **ng/mL**—nanogram/milliliter; **LOD**—limit of detection; and **CFU/mL**—colony-forming units per milliliter.

## 5. Recent Advances in CQDs@MOFs for Detection Applications of Food Contaminates

Advances in food detection applications have been fueled by innovations in chemistry, materials science, and technology, focusing on improving food safety, quality, and authenticity. Techniques including fluorescent, electrochemical, and optical sensors are increasingly utilized to detect contaminants, including heavy metals, pesticides, antibiotics, toxins, pathogens, and aromatic compounds, all of which are harmful substances in food samples, allowing for increased sensitivity and the detection of trace analyte concentrations. This review focuses on CQDs@MOFs composites for food detection applications concerning food safety.

### 5.1. Metal Ions

**Figure 2:**
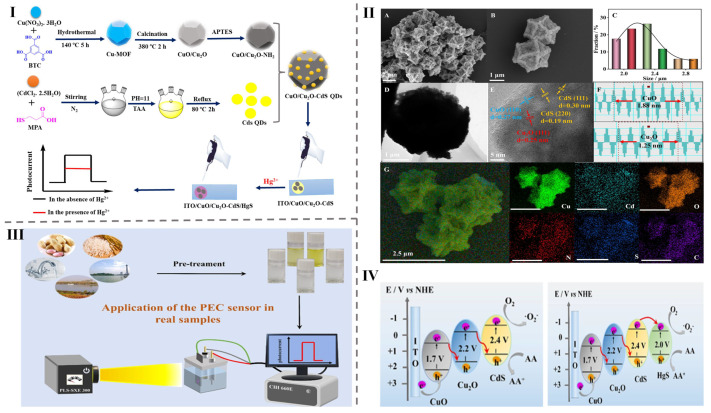
(**I**) Synthesis of CuO/Cu_2_O-CdS/HgS and PEC sensor for Hg^2+^ detection. (**II**) SEM images (**A**,**B**), histogram showing the statistical size distribution (**C**), TEM image (**D**), HRTEM image (**E**), lattice spacing images (**F**), and corresponding elemental mapping images of CuO/Cu_2_O-CdS QDs (**G**). (**III**) Real sample analysis using the PEC sensor for Hg^2+^ detection. (**IV**) Proposed charge transfer mechanisms at ITO/CuO/Cu_2_O-CdS and ITO/CuO/Cu_2_O-CdS/HgS electrodes. Reprinted with permission from [5]. Copyright @2024 Elsevier Ltd.

Metal ions play a vital role in food detection by serving as key components in sensing systems to identify contaminants, nutrients, and other quality indicators [141,142,143,144,145]. Their interactions with food analytes can result in visible color changes, influence fluorescence properties, and act as catalysts in electrodes. These properties are harnessed to detect harmful substances and nutrients in food [146,147,148,149,150,151,152,153,154,155]. For instance, Jain and colleagues created a fluorescent nano-biosensor composite named BNCD/TbMOF@GR5 DNAzyme, which exhibited dual blue–green emissions at 450, 490, and 544 nm, with a sharp peak width of just 3 nm. The material was produced by incorporating water-soluble BNCDs into a luminescent terbium-based metal–organic framework (Tb-MOF) using an in situ hydrothermal synthesis approach. This composite showed excellent fluorescence for detecting lead in water, with a linear range of 0–1000 nM and and LOD value of 5.97 nM [38]. Similarly, the hydrothermal method was used to synthesize and characterize CQDs@ZIF-8 for use in an electrochemical sensor designed to detect multiple metal ions, such as Pb^2+^, Cd^2+^, and Cu^2+^, in tap and river water. The linear detection range was 50 nM^−1^ μM, and the LOD value was 0.04 nM [56]. Additionally, a dual-emission fluorescent sensor, referred to as CDs@Eu-MOFs, was prepared via a hydrothermal synthesis approach. This sensor displayed a sharp emission peak centered at 365 nm with a bandwidth of approximately 3 nm. It was utilized for detecting mercury ions (Hg^2+^) in aqueous samples, demonstrated with a range of 0 to 300 μM and an LOD value of 0.12 nM [57]. Similarly, CuO/Cu_2_O-CdS/HgS photoelectrochemical sensors were fabricated via hydrothermal methods for mercury (Hg^2+^) ion detection in rice, peanuts, and water samples, as illustrated in Figure 2I,III. The structure and morphology of the material were examined using a range of spectroscopic methods, including SEM images (A–B), a histogram depicting statistical size distribution (C), a TEM image (D), an HRTEM image (E), lattice spacing images (F), and corresponding elemental mapping images of CuO/Cu_2_O-CdS QDs (G), as presented in Figure 2II. The charge transfer mechanisms at the ITO/CuO/Cu_2_O-CdS and ITO/CuO/Cu_2_O-CdS/HgS electrodes were proposed for real sample detection, as depicted in Figure 2IV. These sensors exhibited a linear detection range of 0.5 pM to 2 μM with an LOD of 0.00011 nM [5]. In another study, a hydrothermal synthesis approach was employed to create a MOF/CdTe quantum dot composite designed for the fluorescent detection of Hg^2+^ and Cu^2+^ ions. The material exhibited a fluorescence emission shift from the orange–red to the blue region, covering a range of 425 to 605 nm. This composite effectively combines the tunable photoluminescence and adaptable characteristics of CdTe QDs with the structural and functional benefits offered by the MOF framework. The sensor achieved LOD values of 0.6996 nM for Hg^2+^ and 0.8268 nM for Cu^2+^ in real samples like lake water, fruit juice, and red wine. Notably, the red wine detection showed promising results [58].

Furthermore, a dual-mode “turn-on/off” fluorescent sensor, CDs@ZIF-90, was developed, showing enhanced emission at 453 nm with Al^3+^ and quenching with Hg^2+^. It enabled sensitive detection of both ions in Yellow River water, with wide linear ranges (1–200 μM for Al^3+^ and 0.05–240 μM for Hg^2+^) and LOD values of (810 nM for Al^3+^ and 19.6 nM for Hg^2+^) [59]. For example, E-CDs@ZIF-8, synthesized hydrothermally, enables rapid (>1s) “turn-on” fluorescence detection of Cu^2+^ (399–405 nm) with a 3.48 nM LOD well below U.S. EPA limits. The method was validated in zebrafish and water samples, showing 97–112% recovery [60]. Asadi et al. also introduced an environmentally friendly synthesis approach for PEG-ZnS QDs@ZIF-67, which was later utilized as a fluorescent sensor to detect Cu^2+^ ions in aqueous samples. The sensor exhibited an emission intensity at 420 nm, a detection range of 3 to 500 nM, and an impressive LOD value of 0.96 nM [61]. Furthermore, the CsPbBr_3_/HZIF-8 composite was synthesized via in situ growth at room temperature and showed green emission at 510 nm with a width of 25 nm. It served as an on–off–on luminescent sensor for detecting Cu^2+^ and melamine in water. Detection was linear from 3 to 500 nM (Cu^2+^) and 30 to 1500 nM (melamine), and the LOD values were 4.66 nM and 2.64 nM, respectively [62]. The overall results are summarized in Table 4. Integrating CQDs@MOFs hybrids into sensing platforms has transformed metal ion detection with enhanced sensitivity, selectivity, and speed. These innovations enable real-time, precise food safety monitoring. Future sensors will focus on miniaturization, wearable integration, and AI-driven analysis for broader use in healthcare and environmental fields.

While the reviewed studies demonstrate remarkable advancements in the development of CQDs@MOFs-based sensors for metal ion detection, their practical implementation in real-world food systems remains underexplored. Most investigations focus on aqueous environments such as tap water, river water, or model solutions. However, real food matrices such as dairy, meat, or processed foods introduce complex interferences (e.g., fats, proteins and varying pH levels) that can affect sensor sensitivity, stability, and selectivity. Matrix effects, sample preparation challenges, and sensor reproducibility must be addressed for reliable deployment in the food industry. For instance, sensors that showed excellent performance in wine or fruit juice still require validation across a broader range of food types. Therefore, future work should emphasize sensor robustness in diverse and complex food matrices, regulatory compliance, and integration into user-friendly, portable, or even wearable formats for field or in-line use in food quality monitoring systems.

### 5.2. Pesticides

**Figure 3:**
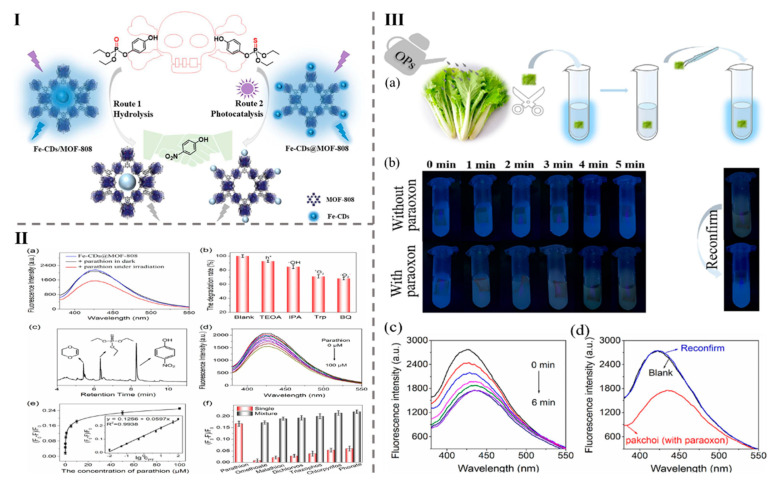
(**I**) Schematic illustration of paraoxon degradation and detection by Fe-CD/MOF-808 (Route 1) and parathion by Fe-CD@MOF-808 (Route 2). (**II**) (**a**) Fluorescence spectra of Fe-CDs@MOF-808 before and after incubation with parathion in the dark and under 365 nm LED irradiation. (**b**) Degradation rate of parathion catalyzed by Fe-CDs@MOF-808 in the presence of different reactive oxygen species (ROS) scavengers. (**c**) GC-MS analysis of degradation products of parathion. (**d**) Fluorescence spectra of Fe-CDs@MOF-808 incubated with varying concentrations of parathion under 365 nm LED irradiation. (**e**) Calibration plot of (F_0_-F)/F_0_ at 425 nm versus parathion concentration. (**f**) Selectivity and anti-interference study of Fe-CDs@MOF-808 for parathion detection. (**III**) (**a**) Schematic representation of organophosphate (OP) detection in pakchoi. (**b**) Fluorescence images showing Fe-CDs/MOF-808 with pakchoi in the absence (left) and presence (right) of paraoxon, confirming the complete degradation of paraoxon in pakchoi. (**c**) Fluorescence spectra of Fe-CDs/MOF-808 with pakchoi in the presence of paraoxon at different time intervals. (**d**) Fluorescence spectra of Fe-CDs/MOF-808 with pakchoi after being removed from the solution for 5 min. Reproduced with permission from [11]. Copyright@ 2023 Elsevier B.V.

Innovations in pesticide detection in food have been groundbreaking, primarily driven by innovations in analytical techniques [156,157,158]. Among these, CQDs and MOFs have played a pivotal role by significantly enhancing signal sensitivity and enabling the development of portable detection devices with high selectivity for specific pesticides [159,160]. For example, emerging technologies are advancing detection methods, such as a room-temperature-fabricated PEC sensor based on M-TiO_2_-CdTe QDs/CdSQDs for CAP monitoring. It offers a 1–140 nmol L^−1^ linear range, 0.14 nM detection limit, and 390 nm emission. The sensor accurately quantified CAP in milk, with recoveries of 96.3–106% [66]. Another example is the room-temperature synthesized N-CDs@Eu-MOF@MIP composite, which enabled sensitive fluorescence detection of malathion (LOD: 50 nM, range: 1–10 μM, λ_-_em: 430–616 nm, and 3 nm bandwidth). It obtained recoveries ranging from 93.0% to 99.3% in samples of lettuce, tap water, and soil. A smartphone-based method detected 2–7 μM malathion with an LOD value of 1.45 μM and a linear response (y = 0.1882x + 0.3166, R^2^ = 0.984). A visible fluorescent shift from red to blue confirmed malathion’s presence [67]. For instance, Ma et al. (2023) synthesized two composite materials, Fe-CDs/MOF-808 and Fe-CDs@MOF-808, at room temperature and applied this for detecting the pesticides paraoxon and parathion, as shown Figure 3I. These nanocomposites exhibited selective and sensitive fluorescence quenching, caused due to the internal filtering effect, with the 4-nitrophenol (4-NP) emission range peaking at 425 nm, as depicted in Figure 3II. Specifically, Fe-CDs/MOF-808 demonstrated a wide linear detection range for paraoxon (0.001–360 μM) and an LOD value of 0.3 nM. In contrast, Fe-CDs@MOF-808 exhibited a linear range of 0.01–100 μM for parathion with an LOD value of 3.3 nM. These materials were successfully tested on real samples of pakchoi and water, underscoring their potential for nanocomposite-based detection and detoxification applications in food safety, as illustrated in Figure 3III [11].

Furthermore, the fluorescent OPCD@UiO-66-NH_2_ composite, synthesized hydrothermally, detected quinalphos with high sensitivity (LOD: 0.3 nM) across 0–16 μM, showing a 425 nm emission peak. Cu^2+^-induced fluorescence quenching, and real sample tests in tomato juice and rice confirmed its effectiveness [12]. For instance, an electrochemical sensor was constructed on a screen-printed electrode using a N-CQDs@UiO-66-NH_2_ composite synthesized via a reflux method. It enabled a highly selective carbendazim detection range and an LOD value of (0.02–126 µM, 5.8 nM), with over 95% selectivity. Recovery rates reached 96% in vegetables and 97% in water samples [27].

Additionally, the CdS-Sm-BDC-g-C_3_N_4_-5 composite was synthesized at room temperature for malathion sensing. The sensor demonstrated excellent sensitivity at 25 μA μM^−1^, with an LOD value of 7.4 nM and a linear response range from 3.0 to 15.0 × 10^−8^ M, exhibiting a strong correlation coefficient (R^2^ = 0.996). It performed well in real cabbage sample tests with recovery rates of 86.4–107.6%. The modified electrode offered good stability, reproducibility, and cost-effectiveness [28]. The overall results are summarized in Table 4. New technologies offer the potential of hybrid CQDs and MOFs to enable real-time, on-site pesticide detection, improving food safety. These nanotech innovations support faster decisions and stricter regulatory compliance. Portable, multi-analyte sensors offer accurate, rapid, and cost-effective monitoring. As these technologies evolve, they will integrate into everyday food safety practices, empowering consumers and regulators with better tools for ensuring safe food. While many CQD–MOF-based sensors have demonstrated impressive analytical performance under laboratory conditions, their practical application in real food matrices remains a key challenge. Food samples often present complex environments with multiple interfering substances that may affect sensor sensitivity, selectivity, and stability. Sample preparation steps such as extraction, filtration, or dilution are often necessary to maintain accuracy, which can limit the sensor’s portability and on-site usability. Moreover, matrix effects such as pH variation, presence of proteins or fats, and natural fluorescence can complicate the interpretation of results. Despite these challenges, some studies have shown promising recoveries in diverse matrices such as milk, lettuce, cabbage, tomato juice, and rice. Continued research should focus on enhancing matrix tolerance, simplifying sample processing, and validating sensors under field conditions to support real-world integration of these innovative sensing platforms.

### 5.3. Antibiotic

**Figure 4:**
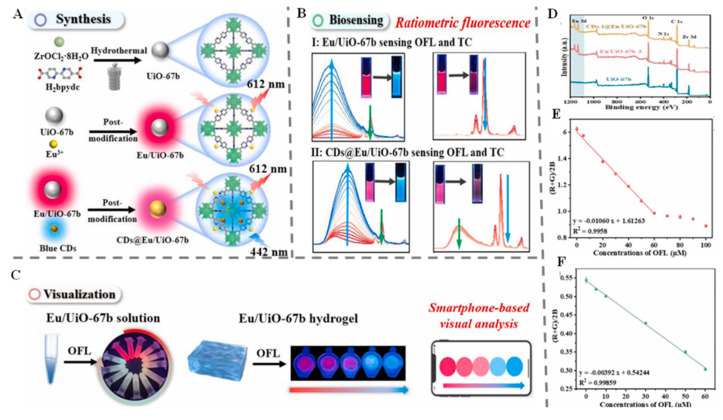
(**A**) Schematic representation of the preparation process for Eu/UiO-67b and CDs@Eu/UiO-67b, (**B**) sensing mechanism for OFL and TC detection, and (**C**) visual detection application using a smartphone. (**D**) XPS survey spectra with inset images showing corresponding photographs. (**E**) and (**F**) display the fitting curves correlating OFL concentration with the color change ratio (R + G)/2B in solution and hydrogel, respectively. Reproduced with permission from [20]. Copyright@ 2024 Elsevier B.V.

Advancements in antibiotic detection in food have become crucial in addressing growing food safety concerns and combating the misuse of antibiotics in food production. Modern techniques now emphasize rapid, accurate, and sensitive detection of antibiotic residues, employing methods such as electrochemical, fluorescence, and colorimetric sensors for real-time monitoring [161,162,163,164,165,166,167,168,169,170,171]. Building on this, dual-functional fluoroprobes (CDs@Eu/UiO-67b) were synthesized hydrothermally, enabling tunable red-to-blue emissions (442–612 nm) for detecting ofloxacin and tetracycline via an internal filtering effect. This ratiometric assay achieved detection limits of 22 nM and 27 nM across 0–60 µM and 0–10 µM ranges, respectively. It showed strong performance in tap water and chicken feed, with recoveries of 98.5–103.7%, demonstrating its potential for real-world antibiotic residue monitoring (Figure 4A–F) [20]. The CdTeQDs@ZIF-8 composite, synthesized at room temperature, enables the ratiometric fluorescent detection of chlortetracycline (CTC) via green and red emissions (521–672 nm) through the inner filtration effect. It offers an LOD value of 37 nM, which is 17 times below the CTC residue limit in animal food (626 nM). The sensor worked effectively, and CTC was detected in basa fish and milk, with recovery rates of 91.0–110.0% being observed, demonstrating its speed, sensitivity, and recyclability for food safety monitoring [29].

Similarly, Liu et al. synthesized a CDs@UiO-66-NH_2_ composite by ultrasound-assisted functionalization of CQDs from fragrans with MOFs. The material enabled simultaneous detection of tetracycline (150 nM) and norfloxacin (870 nM), with emission at 328 nm. It performed well on real samples of water, milk, and soil [68]. In another example, a CD@MIP composite was synthesized under reflux conditions and applied for the detection of tetracycline in milk samples. The material exhibited fluorescence quenching with an emission peak at 450 nm and a width of 5 nm. Importantly, the detection was effective over a concentration range of 0–400 μmol L^−1^. Furthermore, the quantum yield of the CD@MIP composite was found to be 12.75%, with a 3σ LOD value of 590 nM [69]. Another noteworthy example is the Ce, N-CDs@ZIF-67@MIP composite, synthesized at room temperature, which acts as a fluorescent sensor for oxytetracycline with bright blue fluorescence (445 nm) and a high quantum yield (33.69%). It offers sensitive detection within a 0.05–20 μg/mL range and a low limit of 15.13 nM, enabling effective oxytetracycline analysis in milk samples [70]. Similarly, the His-GQDs-Ser@MOF composite was synthesized at room temperature and showed high selectivity and sensitivity for doxycycline detection using a fluorescence sensor (460–618 nm, 5 nm bandwidth). It exhibited two linear detection ranges (0.003–6.25 μM and 6.25–25 μM) with an LOD value of 1.8 nM. The probe’s practical application was validated by analyzing spiked milk samples, with recoveries between 97.39% and 103.61% and RSDs ranging from 0.62% to 1.42% [71].

In addition, the g-CDs@UiO-66 composite, prepared through stirring, exhibits excellent optical properties, fluorescence stability, and structural robustness in aqueous solutions. Combining the optical features of g-CDs and UiO-66, it serves as an effective probe for detecting norfloxacin with an emission range of 446–530 nm, detection range of 1–8 μM, and LOD value of 82 nM. The composite exhibits excellent selectivity and sensitivity, making it suitable for detecting norfloxacin in food samples such as milk and pork. This study highlights advancements in CD-MOF-composite sensing for pesticide residue detection in food [72]. The summarized findings are presented in Table 4. The effective implementation of these detection systems demonstrates their strong potential for enhancing food safety by enabling real-time and efficient monitoring of antibiotic residues in food items. Recent advances in CQD–MOF composite sensors have shown promising applicability in complex food matrices such as milk, pork, fish, and feed. As summarized in Table 4, many systems have achieved high recovery rates (e.g., 91.0–110.0% in basa fish and milk and 97.39–103.61% in spiked milk), low limits of detection, and good reproducibility, indicating their strong potential for real-world use. However, transitioning these sensors from laboratory demonstrations to practical, field-ready tools involves several critical challenges. These include matrix interferences due to the complex chemical composition of food samples, variability in sample preparation methods, sensor stability under varying environmental conditions, and the need for scalable, low-cost fabrication processes. Furthermore, regulatory acceptance requires thorough validation under standardized protocols.

### 5.4. Mycotoxins

**Figure 5:**
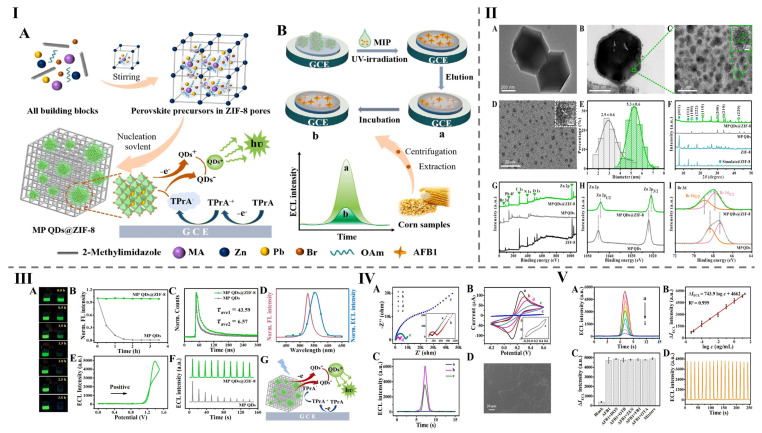
(**I**) Schematic illustration of the MP QDs@ZIF-8-based molecular imprinting ECL sensor for AFB1 detection in corn samples. (**A**) Synthesis process and proposed ECL reaction mechanism of MP QDs@ZIF-8 nanocomposites. (**B**) Signal responses of the AFB1-imprinted ECL sensor throughout the detection process. (**II**) (**A**–**I**) TEM and HRTEM images, size distribution, XRD patterns of simulated, XPS full spectra, and high-resolution XPS spectra of ZIF-8, MP QDs, and MP QDs@ZIF-8. (**III**) (**A**–**G**) Optical images of MP QDs and MP QDs@ZIF-8, along with fluorescence intensity variations over time, decay curves, and time-dependent evolution of MP QDs and MP QDs@ZIF-8 composites. Fluorescence and ECL wavelength spectra of MP QDs@ZIF-8 with optical filters, ECL-potential curve, and ECL-time responses of MP QDs@ZIF-8 and MP QDs. A schematic representation of the proposed ECL reaction mechanism is also included. (**IV**) (**A**–**D**) EIS plots and CV curves of MP QDs@ZIF-8/GCE and AFB1-imprinted MP QDs@ZIF-8/GCE, along with the ECL response of the proposed AFB1-imprinted sensor in PBS containing 0.01 M TPrA. Additionally, an SEM image showcasing the surface morphology of the AFB1-imprinted ECL sensor is presented. (**V**) (**A**–**D**) ECL signals of the eluted AFB1-imprinted sensor after rebinding in various concentrations of AFB1 solutions, along with the calibration curve for AFB1 detection. Δ*I*_ECL_ responses of the eluted AFB1-imprinted ECL sensor following incubation in blank solution, 10 ng/mL of DON, OTB, ZEN, FB1, or OTA as interferences, and 1 ng/mL AFB1 solution as a target, including a mixture of all interferences with AFB1. Additionally, the ECL response of the imprinted sensor incubated with 0.1 pg/mL AFB1 is shown after continuous CV scans for 17 cycles. Reprinted with permission from [8]. Copyright @2022 Elsevier Ltd.

Mycotoxins are toxic secondary metabolites produced by various fungal species, and pose significant health risks to humans and animals. They can contaminate food and feed at multiple points throughout the production and supply chain, making their identification and elimination a critical concern worldwide [172,173,174,175,176,177,178,179]. In this context, this study investigates the design, synthesis, and application of CQDs@MOFs composites for the detection of mycotoxins in food samples, with particular emphasis on their sensing mechanisms, performance metrics, and potential for practical implementation in food safety monitoring [180,181,182,183,184,185,186]. For instance, the MP QDs@ZIF-8 composite was fabricated at room temperature and employed in an electrochemical sensor for detecting Aflatoxin B1 (AFB1), as shown Figure 5I. The characterization of ZIF-8, MP QDs, and MP QDs@ZIF-8 was carried out using various techniques, including TEM and HRTEM imaging, particle size distribution analysis, and XRD pattern analysis of the simulated structures, as well as by studying both full and high-resolution XPS spectra, as illustrated in Figure 5II. This composite exhibited an emission peak at 528 nm with a bandwidth of 21 nm. The sensor demonstrated exceptional selectivity and ultra-sensitivity, achieving an LOD value of 0.0000035 nM and a detection range for AFB1 quantification spanned from 11.55 fg/mL to 20 ng/mL, as demonstrated Figure 5III. The EIS plots and CV curves of MP QDs@ZIF-8/GCE and AFB1-imprinted MP QDs@ZIF-8/GCE, along with the ECL response of the proposed AFB1-imprinted sensor in PBS containing 0.01 M TPrA, are presented in Figure 5IV. Furthermore, the successful recovery results from corn samples confirmed the sensor’s accuracy and real-world potential for detecting AFB1. Overall, this study introduces an innovative strategy for developing efficient electrochemical sensing systems to enhance food safety, as illustrated in Figure 5V [8].

In a similar approach, an antibody-conjugated MoS_2_/UiO-66-NH_2_ composite was created through a microwave-assisted method for the sensitive electrochemical detection of aflatoxin M1 (AFM1). This sensor exhibited a detection range of 0.2–10 ng/mL and an LOD value of 0.06 ng/mL. Furthermore, its practicality was demonstrated by successfully detecting AFM1 in spiked milk samples. Notably, this approach can be adapted for the detection of other aflatoxins, such as AFB1 [21]. Building on this, the synthesis of the NU66@QD-ICA composite under room-temperature conditions resulted in a fluorescent sensor with an emission range of 400–670 nm. This sensor facilitated the sensitive detection of various toxins, including aflatoxin B1, fumonisin B1, deoxynivalenol, T-2 toxins, and zearalenone (ZEN) in cereals and feed. The detection limits for these toxins were 0.04, 0.28, 0.25, 0.09, and 0.08 μg/kg, respectively. Moreover, the recovery rates ranged from 82.83% to 117.44%, with variation coefficients between 2.88% and 11.80%, demonstrating the method’s practical reliability [73].

In another example, the SQDs@MOF-5-NH_2_ composite, synthesized via a “bottle-around-ship” solvothermal method, serves as a fluorescent probe for detecting patulin with enhanced fluorescence (645–755 nm). It offers high sensitivity (LOD: 0.000753 ng/mL) and excellent specificity, with low RSDs in assays. Applied to apple juice, it shows strong recovery rates (89.03–107.67%) compared to HPLC results. Despite a 120-min reaction time, its simplified DNA hairpin amplification suggests broader applications for detecting mycotoxins and other biomarkers [74]. Similarly, a hydrothermal method was used to prepare MIP/Au@Cu-MOF/N-GQDs/GCE for electrochemical patulin sensing, showing a broad linear range (0.001–70.0 ng/mL) and an LOD value of (0.0007 ng/mL). The sensor offered excellent selectivity, sensitivity, and reproducibility, with high accuracy (97.6–99.4% recovery) and precision (RSD: 1.23–4.61%) in apple juice. This strategy holds strong potential for other MIP-based sensor applications [75]. The comprehensive findings presented in Table 5 illustrate the advancements in CQDs@MOF-based composites for mycotoxin detection in food samples. This adaptability positions them as promising tools for the broader field of analytical sensing, contributing to enhanced food safety and public health protection.

While the reported CQDs@MOFs-based sensors demonstrate impressive sensitivity, selectivity, and reproducibility in controlled experimental conditions, practical implementation in real-world food matrices presents additional challenges. Complex sample matrices such as cereals, milk, and fruit juices often contain interfering substances (e.g., proteins, fats, and polyphenols) that may affect sensor performance by causing matrix effects or signal suppression. Therefore, effective sample pretreatment and matrix-matching strategies are critical for ensuring analytical reliability in real applications. Additionally, factors such as sensor stability under varying storage conditions, reproducibility across production batches, and scalability of sensor fabrication must be addressed to facilitate commercialization. Despite these challenges, recent studies have shown promising results, with high recovery rates and low RSD values in spiked food samples-indicating that the transition from lab to field is feasible. Continued development toward miniaturized, portable sensor platforms and integration with digital readout systems may further support the deployment of CQDs@MOFs sensors in real-time food safety surveillance.

### 5.5. Pathogens

**Figure 6:**
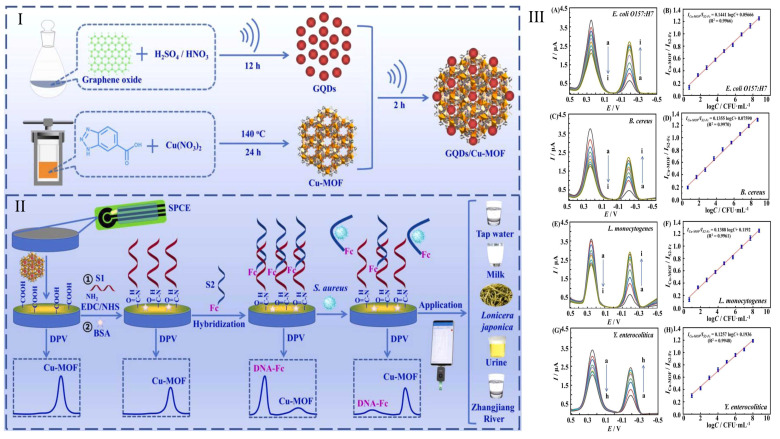
(**I**) Synthesis of the GQDs/Cu-MOF nanocomposite, (**II**) development of a GQDs/Cu-MOF nanocomposite-based ratiometric electrochemical aptasensor for detecting *S. aureus* in tap water, milk, *Lonicera japonica*, urine, and the Zhangjiang River. (**III**) DPV responses for varying concentrations of *E. coli* O157:H7 (**A**), *B. cereus* (**C**), and *L. monocytogenes* (**E**): a to i represent 5.0 × 10^0^, 5.0 × 10^1^, 5.0 × 10^2^, 5.0 × 10^3^, 5.0 × 10^4^, 5.0 × 10^5^, 5.0 × 10^6^, 5.0 × 10^7^, and 5.0 × 10^8^ CFU·mL^−1^. DPV responses for different concentrations of *Y. enterocolitica* (**G**): a to h represent 1.0 × 10^1^, 1.0 × 10^2^, 1.0 × 10^3^, 1.0 × 10^4^, 1.0 × 10^5^, 1.0 × 10^6^, 1.0 × 10^7^, and 1.0 × 10^8^ CFU·mL^−1^. A linear correlation between *I*_Cu-MOF_/*I*_S2-Fc_ and the logarithm of CFU·mL^−1^ was observed for foodborne pathogens: *E. coli* O157:H7 (**B**), *B. cereus* (**D**), *L. monocytogenes* (**F**), and *Y. enterocolitica* (**H**). Reproduced with permission from [76]. Copyright@ 2024 Elsevier B.V.

Pathogens are harmful microorganisms such as bacteria, viruses, and parasites that can cause illness through contaminated food. Common examples include *Salmonella*, *E. coli*, *Listeria*, and *Norovirus*. They may lead to symptoms like diarrhea, vomiting, fever, or even severe complications. Ensuring food safety involves detecting and controlling these pathogens in the supply chain. This review highlights CQDs@MOFs as a novel sensing platform for the swift and selective identification of pathogenic bacteria in food. The unique fluorescence of CQDs, integrated with the porous architecture of MOFs, allows for highly sensitive detection even in complex food environments [187,188,189,190,191,192]. By functionalizing the material with specific targeting agents, such as antibodies or aptamers, this hybrid system addresses critical needs in food safety monitoring by offering precise pathogen recognition [193,194,195,196,197,198,199]. For instance, Lin et al. reported the synthesis of a GQDs/Cu-MOF nanocomposite using an ultrasonication method, as shown Figure 6I. This composite was employed for the detection of *Staphylococcus aureus* (*S. aureus*) via electrochemical aptasensors. The system demonstrated remarkable sensitivity with an LOD of 0.97 CFU/mL, alongside excellent stability, specificity, and a broad linear detection range of 5.0 × 10^0^ to 5.0 × 10^8^ CFU·mL^−1^. In addition, the aptasensor was effectively used to detect *S. aureus* in various samples, such as tap water, milk, Lonicera japonica, urine, and water from the Zhangjiang River. Additionally, the design of this aptasensor is highly adaptable, allowing for the detection of other foodborne pathogens, as illustrated in Figure 6II. Furthermore, the detection ranges and LODs for various pathogens were as follows: *E. coli* O157:H7, *B. cereus*, *Y. enterocolitica*, and *L. monocytogenes,* as reported in Table 2 and depicted in Figure 6III. These results underscore the versatility of the design, providing valuable tools for early detection of food safety hazards and issuing timely warnings of foodborne diseases. Furthermore, this research provides fresh perspectives on the advancement of novel electrochemical aptasensor technologies [76].

Another example of an rGO-MWCNT/CS/CQD composite was synthesized at room temperature to develop an electrochemical aptasensor for detecting *Acinetobacter baumannii*. This enhanced detection sensitivity and aptamer surface density, improving sensor performance. The aptasensor exhibited a linear range of 10 to 1 × 10^7^ CFU/mL and an LOD value of 1 CFU/mL, and effectively identified *A. baumannii* in serum and milk powder samples [77]. Similarly, the synthesis of DP-CDs/TiO_2_ via hydrothermal methods demonstrated enhanced photocatalytic bacterial deactivation under sunlight irradiation. This composite was employed for the detection of *Vibrio harveyi* using a fluorescence sensor with an emission range of 520–420 nm. In addition, a fluorometric sensor-strip was developed for Fe^3+^ detection and the monitoring of acute hepatopancreatic necrosis disease (AHPND) caused by *Vibrio harveyi* in shrimp farming [78].

In addition, the synthesis of [Zn(HCOO)_3_][C_2_H_8_N]/PEG and N-CQDs@[Zn(HCOO)_3_][C_2_H_8_N]/PEG composites via hydrothermal steps were reported. Their antimicrobial activity against *E. coli* showed strong antibacterial performance under UV light. These composites were presented as cost-effective, biocompatible antimicrobial agents that function without antibiotics [31].

Similarly, CD-Ab-COF was prepared at room temperature and utilized as a fluorescent probe for detecting *E. coli* O157:H7, and showed an emission peak near 365 nm, with the sensor exhibiting a linear detection range of 0 to 10^6^ CFU/mL and an impressive LOD value of 7 CFU/mL. Furthermore, its performance was validated through the analysis of real milk samples [9]. These studies underscore the potential of CQDs@MOFs and carbon-based nanomaterials in advancing food safety diagnostics by enabling sensitive, selective, and adaptable pathogen detection to mitigate foodborne outbreaks. Detection parameters and performance metrics are summarized in Table 5. Although CQDs@MOFs-based sensors have demonstrated remarkable sensitivity and selectivity, their real-world application remains a key challenge for practical adoption. Complex food matrices such as meat, dairy, seafood, and ready-to-eat products introduce issues like matrix interference, signal suppression, and intricate sample preparation. Additional concerns include sensor stability under variable storage conditions, manufacturing reproducibility, and minimizing false positives or negatives. Despite these obstacles, several studies have successfully validated CQDs@MOFs sensors in real samples such as milk, river water, and serum, highlighting their promise for broader food safety monitoring. Future efforts should prioritize sensor miniaturization, integration with portable devices, and alignment with regulatory standards to support commercial translation.

### 5.6. Aromatic Compounds

CQDs@MOFs composites offer a promising approach for ensuring food safety by enabling the detection of aromatic contaminants, including 4-nitrophenol, Allura Red, catechol, curcumin, glutathione, malachite green, phenylurea, trilobatin, and triticonazole. These composites operate through fluorescence quenching or enhancement mechanisms, wherein the interaction between the contaminant and the CQDs@MOFs alters its optical signal. This provides a rapid, cost-effective, and non-destructive approach for identifying trace amounts of harmful substances in food samples [200,201,202,203,204,205,206]. Similarly, heterocyclic amines and phenolic compounds from Perilla frutescens seed extract show a correlation between antioxidant capacities and their mitigating effects on volatile compounds during low-temperature ultrasonic marination of coffee leaves and meat [207,208,209,210,211,212]. For example, Yan et al. developed room-temperature-synthesized CDs-MFMIPs for ultrasensitive and selective detection of 4-nitrophenol (4-NP) in food, with a wide detection range (0.05–50 μM) and a low limit of 17.44 nM. The system, featuring magnetic properties and smartphone-assisted visual sensing, enabled rapid, accurate analysis of real samples like tap water, fish, and shrimp. This portable method offers a reliable, practical solution for onsite food safety monitoring [80]. In addition, Esmail et al. developed a CD@MOF nanocomposite at room temperature for detecting Allura Red AC (AR) in food, achieving a 0.30–7.00 nM range and 0.60 nM LOD. It showed high adsorption, reusability, and accurate detection in real samples (candies, syrups, and energy drinks), with AR levels ranging from 2.95 to 2953 mM and 98.44 to 102.41% recovery [81]. For instance, Zhou et al. synthesized CDs@HKUST-1/GCE via a hydrothermal method to create a highly sensitive electrochemical sensor for catechol detection. It exhibited a broad linear range (5.0 × 10^−9^ to 2.5 × 10^−5^ mol/L), LOD value of (3.8 nM), and strong reproducibility and stability in tea sample analysis. This sensor shows promising potential for food analysis and broader analytical applications [82].

Further, Wang et al. synthesized a CDs@MOF-5@Rh-6G composite with distinct dual-emission peaks (435 and 560 nm) under 335 nm excitation, enabling sensitive ratiometric detection of curcumin. The sensor exhibited an LOD value of (15 nM) and a wide linear range (0.1–5 μmol/L), with successful application in detecting curcumin in mustard, curry, and red pepper powders. This study highlights a promising strategy for food quality control using practical and sensitive fluorescence sensing [10]. Furthermore, Jalili et al. developed BYCDs@ZIF-8 nanocomposites for dual-emission detection of glutathione and Cu^2+^ at room temperature. With a single 365 nm excitation, emissions at 565 and 440 nm enabled a visible yellow-to-blue shift under UV light. The method showed high sensitivity, an LOD value of 0.90 nM, and was effective in real samples like grape and cucumber extracts, where it achieved satisfactory and reliable results, demonstrating its potential for practical applications in monitoring food safety [83].

**Figure 7:**
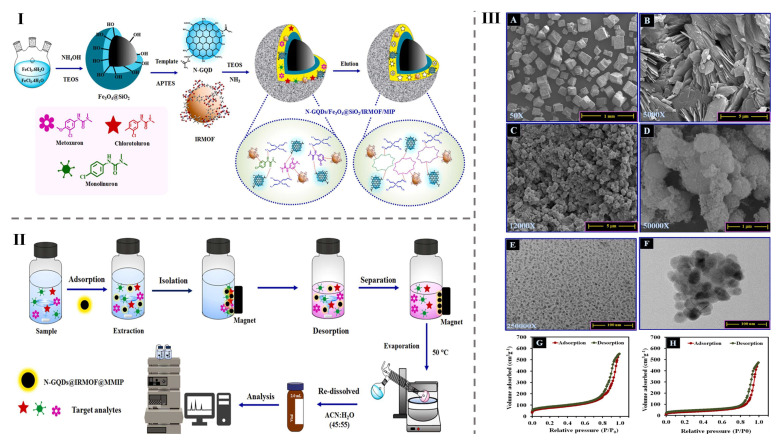
(**I**) Synthesis process of the N-GQDs/Fe_3_O_4_@SiO_2_/IRMOF-1/MIP nano-sorbent, and (**II**) the d-MSPE procedure for phenylurea extraction. (**III**) SEM images of IRMOF-1 (**A**,**B**) and N-GQDs/Fe_3_O_4_@SiO_2_/IRMOF-1/MIP (**C**,**D**). TEM images of N-GQDs (**E**) and GQDs/MIP sorbent (**F**). Adsorption–desorption isotherms for N-GQDs/Fe_3_O_4_@SiO_2_/IRMOF-1/MIP (**G**) and N-GQDs/Fe_3_O_4_@SiO_2_/IRMOF-1/NIP (**H**) nano-sorbents. Reproduced with permission from [85]. Copyright@ 2023 Elsevier Inc.

Additionally, Liu et al. demonstrated that the CDs&ZIF-8@MIPs fluorescent sensor offers high sensitivity and selectivity for malachite green (MG), with a 2.93 nM detection limit and a 20–180 nM linear range. It effectively distinguishes MG from analogs and performs well in real water samples, highlighting its practical potential [84]. For example, Sa-Nguanprang et al. successfully synthesized the N-GQDs@IRMOF-1@MIP composite, IRMOF-1 (isoreticular metal–organic framework-1), MIP (molecularly imprinted polymer), which enabled the development of an exceptionally sensitive detection method for trace amounts of target phenylureas, as shown in Figure 7I. This method exhibited a broad linear detection range of 1.0–150 µg L^−1^, with a remarkably low detection limit of 1.0 µg L^−1^. Furthermore, the method was applied to real-world samples, including tomato, cucumber, radish, and soybean milk, as illustrated in Figure 7II, demonstrating its practical utility for food safety and environmental monitoring. Characterization of the materials was performed through SEM and TEM imaging, providing detailed structural insights into IRMOF-1, N-GQDs, and the GQDs/Fe_3_O_4_@SiO_2_/IRMOF-1/MIP composite. These imaging techniques confirmed the morphology and integrity of the composite at the nanoscale. Additionally, the adsorption–desorption isotherms for N- GQDs/Fe_3_O_4_@SiO_2_/IRMOF-1/MIP (G) and N-GQDs/Fe_3_O_4_@SiO_2_/IRMOF-1/NIP (H) nano-sorbents, presented in Figure 7III, demonstrated the efficient adsorption capacity of the materials. The large specific surface area and well-structured nanoparticle composition of the sorbents significantly enhanced the adsorption performance, leading to an improvement in the overall detection sensitivity and reliability. This novel approach opens up new avenues for the development of highly efficient and versatile detection platforms for food safety applications [85]. Furthermore, Yao et al. developed an AgMOF@N-CD composite at room temperature and used it as an ECL sensor for trilobatin (Tri) detection. The sensor offered a wide linear range (1.0 × 10^−7^ M to 1.0 × 10^−3^ M), LOD value of 5.99 nM, and excellent reproducibility, stability, and anti-interference performance. It also accurately detected Tri in *Lithocarpus polystachyus* Rehd samples, confirming its practical applicability [86].

Similarly, Shokri et al. demonstrated that the B-CDs/P-CDs@ZIF-8 composite, when excited at 385 nm, emits dual peaks at 440 nm and 510 nm. This sensor demonstrated high sensitivity for triticonazole detection with a broad linear range (10–400 nM) and LOD value of (4.0 nM). Its practical application was confirmed by successful analysis of water and fruit juice, highlighting its potential for food safety [87]. The overall results of CQDs@MOFs-based sensing systems, summarized in Table 5, offer high sensitivity, selectivity, and adaptability for detecting harmful aromatic compounds in food. Their unique structural and optical properties enable precise, rapid detection, addressing key challenges in food safety. Continued research promises even more efficient and reliable platforms. This integration highlights their transformative potential in real-world food monitoring and public health protection. While many CQDs@MOFs-based sensing platforms demonstrate exceptional sensitivity and selectivity under laboratory conditions, translating these results to real-world food matrices remains a key challenge. Food samples often present complex, heterogeneous matrices that can interfere with analyte recognition and signal readout due to matrix effects such as pH variability, presence of fats, proteins, or colorants. Moreover, issues like sensor stability in varied storage conditions, reproducibility across batches, and regulatory compliance for use in commercial settings must also be addressed. Encouragingly, several studies have demonstrated successful application in real samples such as energy drinks, seafood, and vegetable extracts—highlighting progress toward practical viability. Nonetheless, standardized sample preparation protocols, matrix-tolerant sensor designs, and integration into portable or on-site testing platforms (e.g., smartphone-assisted readouts) are essential steps toward broad real-world adoption. Addressing these issues through interdisciplinary collaboration will be critical to bridging the gap between laboratory innovation and field implementation.

## 6. Challenges and Future Perspectives

Scaling up the production of CQDs@MOFs with consistent size, morphology, and properties is challenging, requiring precise control over reaction conditions and costly reagents. Both CQDs and MOFs are sensitive to moisture, light, and extreme temperatures, which can degrade their functionality. Moreover, non-specific interactions with environmental components may reduce sensing accuracy. Achieving high selectivity for specific analytes in complex food matrices is difficult, and detecting low concentrations while avoiding interference remains a challenge. Concerns about the potential release of CQDs or MOFs into food products raise toxicity and regulatory issues. Ensuring safety for human consumption is crucial. Additionally, integrating CQDs@MOFs into portable, user-friendly devices for on-site food detection is still underdeveloped. Validating these sensors across diverse food systems adds to the complexity.

The future of CQDs@MOFs composites centers on green, scalable, and energy-efficient synthesis, supported by machine learning and computational modeling for property prediction. Enhancing stability through protective coatings, stabilizers, and tailored functional groups can boost selectivity and sensitivity to specific food contaminants. Integrating nanomaterials like metal nanoparticles and polymers creates multifunctional sensors with improved optical, electronic, and catalytic performance. Research is expanding into detecting emerging contaminants like microplastics, drug residues, and mycotoxins, alongside applications in quality control for beverages, dairy, and perishables. Regulatory approval and safety guidelines are essential for food-related uses.

Long-term environmental and health impact studies, along with the development of affordable, portable, and automated sensors, are critical for broader adoption. Biomass-derived CQDs and recycling strategies enhance sustainability. Industry collaboration is vital to validate these sensors in real-world supply chains. Machine learning also enhances sensor data interpretation, while wireless CQDs@MOFs enable real-time tracking in food storage and distribution systems. These sensors can be embedded in packaging to detect spoilage indicators (e.g., pH, CO_2_, and NH_3_) via color or fluorescence changes and enable controlled antimicrobial release to extend shelf life. Research continues to focus on cost-effective production and scaling to support safe commercialization and regulatory approval.

## 7. Conclusions

The combination of functionalized CQDs with MOFs holds significant promise for revolutionizing food safety and detection applications. However, challenges such as stability, sensitivity, and integration into practical systems must be overcome to ensure their widespread use. With continued advancements in material science, functionalization techniques, and sensing technologies, CQDs@MOFs composites have the potential to play a key role in enhancing food safety monitoring in the future.

## Data Availability

No new data were created or analyzed in this study. Data sharing is not applicable to this article.
